# A machine-learning-based alternative to phylogenetic bootstrap

**DOI:** 10.1093/bioinformatics/btae255

**Published:** 2024-06-28

**Authors:** Noa Ecker, Dorothée Huchon, Yishay Mansour, Itay Mayrose, Tal Pupko

**Affiliations:** The Shmunis School of Biomedicine and Cancer Research, George S. Wise Faculty of Life Sciences, Tel Aviv University, Tel Aviv 6997801, Israel; School of Zoology, George S. Wise Faculty of Life Sciences, Tel Aviv University, Tel Aviv 6997801, Israel; The Steinhardt Museum of Natural History and National Research Center, Tel Aviv University, Tel Aviv 6997801, Israel; The Blavatnik School of Computer Science, Raymond & Beverly Sackler Faculty of Exact Sciences, Tel Aviv University, Tel Aviv 6997801, Israel; School of Plant Sciences and Food Security, George S. Wise Faculty of Life Sciences, Tel Aviv University, Tel Aviv 6997801, Israel; The Shmunis School of Biomedicine and Cancer Research, George S. Wise Faculty of Life Sciences, Tel Aviv University, Tel Aviv 6997801, Israel

## Abstract

**Motivation:**

Currently used methods for estimating branch support in phylogenetic analyses often rely on the classic Felsenstein’s bootstrap, parametric tests, or their approximations. As these branch support scores are widely used in phylogenetic analyses, having accurate, fast, and interpretable scores is of high importance.

**Results:**

Here, we employed a data-driven approach to estimate branch support values with a probabilistic interpretation. To this end, we simulated thousands of realistic phylogenetic trees and the corresponding multiple sequence alignments. Each of the obtained alignments was used to infer the phylogeny using state-of-the-art phylogenetic inference software, which was then compared to the true tree. Using these extensive data, we trained machine-learning algorithms to estimate branch support values for each bipartition within the maximum-likelihood trees obtained by each software. Our results demonstrate that our model provides fast and more accurate probability-based branch support values than commonly used procedures. We demonstrate the applicability of our approach on empirical datasets.

**Availability and implementation:**

The data supporting this work are available in the Figshare repository at https://doi.org/10.6084/m9.figshare.25050554.v1, and the underlying code is accessible via GitHub at https://github.com/noaeker/bootstrap_repo.

## 1 Introduction

To estimate the reliability of individual clades in an inferred phylogenetic tree, it is a common practice to employ both parametric and nonparametric approaches. Felsenstein (1985) proposed using the nonparametric bootstrap (Efron and Tibshirani 1993), in which resampling of alignment columns is used to generate a set of pseudoalignments. From each such pseudoalignment, a pseudotree (also called a bootstrap tree) is generated. The bootstrap support of each branch (a bipartition of the unrooted tree) is defined as the fraction of bootstrap trees in which this bipartition exists. While bootstrap computations have become the standard in any phylogenetic analysis, this nonparametric bootstrap necessitates the repetition of the tree-search process numerous times, a task that demands a substantial amount of computational time, especially in the case of maximum-likelihood-based tree-searches. Hence, state-of-the-art tree-search software incorporate approximate versions of the standard bootstrap approach, e.g. ultrafast bootstrap in IQTREE (Minh *et al.* 2013, Hoang *et al.* 2018) and rapid bootstrap in RAxML (Stamatakis *et al.* 2008). The primary advantage of the bootstrap approach lies in its nonparametric nature, i.e. it does not rely on any distributional assumptions that could potentially be incorrect. However, Efron *et al.* (1996) showed that Felsenstein’s bootstrap provides only a first-order approximation to the actual support values and may become poor depending on the curvature of the tree-space. To address this limitation, Efron proposed conducting additional second-level bootstrap replications, which would be utilized to correct the standard bootstrap score, accounting for the curvature of the tree-space. The number of second-level bootstrap replicates should be substantially greater than that of the first-level approximation, demanding significant computational resources. Indeed, this correction is not implemented in any common phylogenetic software.

Fast parametric and semiparametric alternatives to the bootstrap were developed and incorporated in state-of-the-art tree search software. The aLRT test (Anisimova and Gascuel 2006) was suggested as a fast and robust approximation to the standard likelihood ratio test. The test statistic is computed as twice the difference between the log-likelihood of the best topology to the log-likelihood of its best nearest neighbor interchange (NNI) topology around the branch in question. This statistic is then compared to a mixture distribution composed of $\chi_{0}^{2}$ and $\chi_{1}^{2}$ components. The aBayes test (Anisimova *et al.* 2011) is a Bayesian modification of the aLRT statistic, which approximates the posterior probability of a configuration around the branch in question based on the log-likelihood scores of the three possible NNI configurations. In both aLRT and aBayes tests, optimization is performed solely for the branch in question and its four adjacent branches, thus reducing running-time. The SH-like local branch support test is another variation of the aLRT test, which relies on resampling the data following the SH test (Shimodaira and Hasegawa 1999).

Different branch support values have distinct interpretations and demonstrate varying sensitivity to model misspecifications. For instance, bootstrap supports are generally more conservative compared to posterior probability values (Douady *et al.* 2003). Anisimova *et al.* (2011) compared Bayesian posterior probabilities, parametric support values, and the nonparametric bootstrap, based on simulations and empirical data analysis. For a given support threshold α, they calculated the false positive rate (FPR), false negative rate (FNR), and the Matthews correlation coefficient (MCC) by labeling branches as “correct” when the support value is above a specified threshold. The authors demonstrated that for the task of binary prediction, both aLRT and aBayes support values significantly outperformed nonparametric approaches. The authors asserted that, despite the desirability of a probabilistic interpretation for branch support, none of the mentioned methods for assessing such support can provide it, even when the underlying model is correctly specified.

In this work, we propose employing machine-learning algorithms to develop a new branch-support score. The score relies on multiple features extracted from the multiple sequence alignment (MSA) and the reconstructed maximum-likelihood tree. The machine-learning model was optimized on extensive training data. Analyzing test data, we demonstrate that this score is more accurate than previously suggested scores. This is also true under model misspecification conditions. One of the limitations of previously developed branch support scores is their interpretation. Our score is calibrated to represent the probability of each bipartition to exist in the true tree. We demonstrate that the probabilities obtained by our model are more accurate than those obtained using the widely used support values provided by state-of-the-art phylogenetic software. Finally, our trained model is substantially faster than the classic Felsenstein’s bootstrap.

## 2 Materials and methods

### 2.1 Bipartition inference as a machine-learning classification task

We conceptualize branch support estimation as a classification task (as in Anisimova *et al.* 2011). We classify only bipartitions that are found in the inferred maximum-likelihood tree. The *y* label for each such bipartition is 1 when the bipartition is found in the true tree, i.e. the tree that generated the data (and *y *=* *0 otherwise). The true label is known from simulated data. We aim to predict this label based on a set of features extracted for each bipartition, e.g. the branch length associated with this partition in the maximum-likelihood tree. Independently generated labeled data are also used to evaluate performance. The trained classifier outputs a score for each bipartition. This predicted label, $\hat{y}$, is based on a cutoff value, *C*. If the machine-learning score is higher than *C*, $\hat{y}=1$ ($\hat{y}=0$ otherwise). We define true-positive predictions as those with $\hat{y}=y=1$. Such bipartitions were supported based on the machine-learning classifier, and are also found in the true tree. Similarly, false positive predictions ($\hat{y}=1,\;y=0$) are those bipartitions of the maximum-likelihood tree that were supported by the machine-learning algorithm, yet they do not appear in the true tree. This allows us to compute confusion matrices for our classifier. We used *C = 0.5* for computing confusion matrices. One can consider the classic Felsenstein’s bootstrap methodology as such a machine-learning algorithm, in which there is only one feature (the bootstrap score), and no training is performed. Notably, training of a classifier based on a single feature such as the Felsenstein’s bootstrap should not change the ranking of results, and thus should not have any significant effect on performance measurements such as the area under the ROC curve (AUC).

### 2.2 Branch support methods without machine-learning

Assume that we use a specific program for tree inference and for branch support, e.g. IQTREE (Minh *et al.* 2013) with its ultra-fast bootstrap estimate. We evaluated the performance of this branch support methodology within a classification scheme. To this end, we used a test database of true trees along with their corresponding set of inferred trees. Internal branches (bipartitions) in these inferred trees are associated with ultra-fast bootstrap values. True trees were generated using simulations (see below). This allowed us to estimate confusion matrices and from these confusion matrices, performance was evaluated using AUC, MCC, FPR, FNR, and F1 score.

The branch support values are often generated via programs that implement specific maximum-likelihood-based heuristic approach. All combinations of tree inference and branch support methods that were evaluated, are listed in Table 1.

**Table 1.** Several branch support methods implemented in current tree search software.

Program	Branch-support method	References
RAxML-NG	Standard Felsenstein’s bootstrap	(Kozlov *et al.* 2019)
RAxML-NG	Transfer bootstrap expectation	(Lemoine *et al.* 2018, Kozlov *et al.* 2019)
IQTREE	Ultrafast bootstrap	(Minh *et al.* 2013, Hoang *et al.* 2018)
IQTREE	aLRT test	(Anisimova and Gascuel 2006)
IQTREE	aBayes test	(Anisimova *et al.* 2011)
FastTree	SH-like test	(Price *et al.* 2010)

### 2.3 A novel machine-learning approach for branch support

Various features can be extracted for each branch in question, e.g. its length and the lengths of the surrounding branches. We thus examined whether using multiple features can provide accurate classifications (See below for a list of features). Following feature selection, we generated a trained machine-learning classifier and evaluated its performance. We generated training data that include a large set of true trees, inferred trees, whether each branch in the inferred tree is in the true tree, and their associated branch support scores. The following classifiers were considered: Gradient Boosting Trees, Random Forest, and Neural Networks (see below).

### 2.4 Interpreting branch support scores as probabilities

Ideally, the branch support values should reflect probabilities, thus providing meaningful and intuitive interpretation. For example, we would like that on average, a branch support of 70% would signify that the branch is correctly placed in the true tree in 70% of the cases. We used the term calibration accuracy (see definition below) as a measure of how well a specific branch score corresponds to probabilities. We compared calibration accuracies of the developed machine-learning classifier as well as standard branch-support values. As we show below, the developed classifier outperforms previous approaches, both in terms of classification and calibration accuracy.

### 2.5 Simulation of MSAs

We generated train and test data as follows. Dataset1 (DS1) included 6000 simulated MSAs with 100–10 000 sites and between 30 and 1000 taxa. Each such MSA was simulated along a different tree topology using AliSim (Ly-Trong *et al.* 2022), based on the script provided in the Github repository of RAxML-grove (Höhler *et al.* 2022). The 6000 different trees were randomly sampled from the RAxML-Grove database, which contains trees derived from empirical datasets (Höhler *et al.* 2022). Each such MSA was simulated using the DNA model associated with that tree in RAxML-Grove. DS1 was further divided to train (DS1.a) and test data (DS1.b). Specifically, 70% of the MSAs in DS1 were randomly selected to form the training data and the remaining 30% were used as test data. Each MSA and its associated bipartitions derived from the maximum-likelihood tree were either in the train data or in the test data, never in both. We ensured that the number of sequences included in the MSAs is similar between the train and test set by dividing the data to five equally sized bins, and sampling 70% of the MSAs from each bin for the training data. Dataset2 (DS2) included 750 additional MSAs simulated along 250 independent trees sampled from RAxML-Grove database. DS2 served as validation data, specifically to assess the impact of model misspecification. DS2 comprise three datasets: DS2.a, in which there is no model misspecification (i.e. we used the model assigned by RAxML-Grove), was simulated along the 250 independent trees similar to DS1; DS2.b was simulated using the same trees as DS2.a but using the Jukes and Cantor (JC) model (Jukes and Cantor 1969) in all simulations; DS2.c was also simulated along the same 250 trees as DS2.a, but using the GTR+F + G+I model (Rodríguez *et al.* 1990). The trees, the models, and the MSAs have been deposited in the Figshare repository at https://doi.org/10.6084/m9.figshare.25050554.v1.

### 2.6 Tree-searches and bootstrap estimates

For each MSA in DS1 and DS2, we performed a tree-search including bootstrap estimates in FastTree (Price *et al.* 2010), RAxML-NG (Kozlov *et al.* 2019), and IQTREE (Nguyen *et al.* 2015). In FastTree, we used the default local support test, which is based on SH test on three alternative topologies (Shimodaira and Hasegawa 1999). In DS1 and DS2.a, tree-searches were conducted assuming the default GTR+CAT model. In DS2.b and DS2.c, tree searches were carried out assuming GTR+CAT and JC+CAT models, respectively. In RAxML-NG, we utilized the default search configuration and the default nonparametric bootstrap configuration, where the number of replicates is automatically determined. Similarly, in IQTREE, we employed the default search configuration. For the bootstrap analysis, we used the ultrafast bootstrap approximation using 1000 replicates, the aBayes test (Anisimova *et al.* 2011), and the parametric aLRT test (Anisimova and Gascuel 2006). In both RAxML-NG and IQTREE, tree searches within DS1 and DS2.a were conducted assuming the same model used for the MSA simulation. Tree searches within DS2.b, DS2.c were conducted assuming GTR+F + G+I and JC models, respectively.

### 2.7 Data preparation

Each analyzed MSA was simulated along a “true” tree (corresponding to a tree obtained from the RAxML-Grove database). The MSA is also associated with a corresponding inferred maximum-likelihood tree, together with its branch support estimates. Each bipartition of an inferred maximum-likelihood tree was labeled with a value of 1 if it is present in the true tree and 0 otherwise. Subsequently, as elaborated in the next section, we extract features from each bipartition, both from the inferred tree and from the MSA. This process results in a dataset in which each row represents a single bipartition, encompassing its corresponding features and a label indicating whether it is present in the true tree. Three such datasets were generated, each one inferred by a different tree search software: RAxML-NG, FastTree, and IQTREE. A machine-learning classifier was trained and evaluated on each such dataset. This was compared to several branch support scores obtained by the corresponding software (Table 1).

### 2.8 Features

For each bipartition within an inferred tree, the following features were extracted: (1) number of sequences; (2) number of MSA columns; (3) number of unique MSA columns; (4) percentage of constant sites; (5) PyPythia MSA difficulty (Haag *et al.* 2022); (6) branch length at the partition site; (7) branch length divided by the mean branch length across the tree; (8) branch length divided by the mean branch length among the four neighboring branches; (9–14) median, 25th percentile, 75th percentile, variance, skewness, and kurtosis of branch lengths distribution in the tree; (15) total tree divergence, i.e. sum of branch lengths; (16) tree deviation from ultrametricity as defined in Tria *et al.* (2017); (17–18) the count and proportion of taxa on the smaller or equal side of the bipartition; (19–20) the cumulative sum of branch lengths and the corresponding fraction on the smaller or equal side of the bipartition; (21–25) the average, minimum, maximum, minimum-to-maximum ratio, and variance of the neighboring branches; (26) the parsimony bootstrap score (fraction of trees in which the bipartition exists across 100 parsimony trees generated by RAxML-NG); (27) the mean transfer distance (Lemoine *et al.* 2018) across these 100 parsimony trees; (28–31) same as (26–27), but considering the average and minimum values for the neighboring bipartitions; (32–33) the fraction of trees in which the bipartition exists and the mean transfer distance (Lemoine *et al.* 2018) across the set of suboptimal ML trees obtained by RAxML-NG; (34–37) Same as (32–33), but considering the average and minimum values for the neighboring bipartitions; (38–39) minimal and maximal log-likelihood difference between the current tree and the two NNI neighbors around the bipartition following branch-length optimization. Features 32–37 rely on suboptimal trees. The RAxML-NG software is the only software that returns suboptimal trees and hence these features were extracted only when bootstrap scores were computed using RAxML-NG. All features were computed using dedicated Python scripts.

### 2.9 Machine-learning models

The classification models were built using LightGBM, a decision-tree classifier with gradient boosting (Ke *et al.* 2017), as implemented in the Python package LightGBM. Prior to model fitting, we performed a recursive feature elimination procedure on the train data based on a 5-fold cross-validation using the Python function feature_selection.RFECV in the scikit-learn library (Pedregosa *et al.* 2011). In this approach, features are recursively eliminated by searching for the feature with the least importance (as defined below). The feature is eliminated if removing it increases the performance in cross-validation. The process ends when there is no benefit in removing the least important features. Using the same cross-validation strategy, we optimized the following hyperparameters of the LightGBM model: number of leaves in each tree (25, 50, 100, 200), tree depth (3, 6, 12, infinite), learning rate (0.1, 0.01, 0.001), number of tree estimators (100, 300), and subsample (0.6, 0.8, 1). In the cross-validation procedure, we verified that all partitions associated with the same tree were assigned to the same fold, i.e. the partitions of a single tree were either used for training or testing but not both. To evaluate the importance of each feature, we estimated the average information gain, i.e. the average decrease in entropy when using that feature across the node splits of the decision trees. Aside from LightGBM, two learning algorithms were evaluated: (i) Random-forest, using the implementation of sklearn.ensemble.RandomForestClassifier in the scikit-learn library (Pedregosa *et al.* 2011). The following hyperparameters of the random-forest model were optimized: max depth (3,5,10) and minimal sample split (2,5,10). (ii) Neural network, using the implementation of sklearn.neural_network.MLPClassifier in the scikit-learn library (Pedregosa *et al.* 2011). For the neural network we used two layers, with varying number of neurons in each layer. The numbers of neurons in each layer was considered as a hyperparameter and was chosen using cross-validation from three possible options: ((10,3),(30,5),(50,10)). Two options for the learning rate (alpha) were considered: 0.0001 or 0.05. For calibrating the probabilities obtained from the classification model, we used 5-fold cross-validation based on isotonic regression, using the implementation of calibration. CalibratedClassifierCV in the scikit-learn library (Pedregosa *et al.* 2011).

### 2.10 Performance evaluation

AUC, MCC, FPR, FNR, and F1 score were used as evaluation metrics to assess accuracy across each dataset (DS1.a, DS1.b, DS2.a, DS2.b, DS2.c) using the implementations in the scikit-learn library (Pedregosa *et al.* 2011). The AUC score was also assessed individually for each MSA in the test set (DS1.b), and subsequently, a Wilcoxon signed-rank test was employed to compare our model’s performance with other branch support scores. This test was implemented using scipy.stats.wilcoxon from the SciPy library (Virtanen *et al.* 2020). In addition, we evaluated how well branch support values reflect probabilities. The probabilistic interpretation of the branch support values was depicted using calibration plots and quantified using the expected calibration error (ECE) based on 30 equally spaced bins (Guo *et al.* 2017).

### 2.11 Code availability

The code was implemented in Python version 3.8 and is available through GitHub (https://github.com/noaeker/bootstrap_repo).

### 2.12 Running time analysis

We conducted a performance analysis, comparing the execution times of different branch support approaches on a Linux cluster system running CentOS. The cluster comprises 69 compute nodes, each equipped with a varying number of CPUs ranging from 12 to 256, along with memory configurations ranging from 54 to 754 GB. The evaluation was carried out using a single CPU for consistency.

### 2.13 Empirical data analysis

We applied our machine-learning model to empirical datasets from a database of MSAs curated by Prof. Rob Lanfear, which is available at https://github.com/roblanf/BenchmarkAlignments. From this database, we selected the first 20 DNA MSAs, each containing a maximum of 1000 sequences and 10 000 columns. The corresponding publications are listed in Supplementary Table S3. In addition, we downloaded the “animal dataset” from Yahalomi *et al.* (2020). These data include 78 protein-coding genes from 119 animal species and 10 outgroup species. From this dataset, we selected the first 20 MSAs. For each MSA, we conducted standard tree searches, including bootstrap analysis, using both RAxML-NG and IQTREE. We then compared the machine-learning-based support for each branch within the maximum likelihood tree to Felsenstein’s bootstrap and Transfer Bootstrap support in RAxML-NG, as well as to aLRT and aBayes support in IQTREE.

## 3 Results

### 3.1 Model performance on test data

We formulated the problem of estimating branch support values as a machine-learning classification method. The DS1.a data were used to train the machine-learning algorithm (including cross-validation). The features-based machine-learning model demonstrated high performance on these training data, regardless of the software that was used for tree search (a different machine-learning model was trained for each software). The AUC scores of the various models were 0.974 for the machine-learning models that were trained on trees inferred using IQTREE and RAxML-NG, and 0.972 for trees inferred using FastTree. When the trained model was applied to test data DS1.b, similar results were obtained: IQTREE (0.968), RAxML-NG (0.968), and FastTree (0.963). The small difference in performance between the train and test data (<0.009 AUC scores for all programs) indicates little to no overfitting of the model. Moreover, the very small differences in AUC among the three programs suggest that the impact of the tree search algorithm on the inferred branch-score values is minimal. Similar results are obtained when considering other evaluation metrics such as MCC, FPR FNR, and F1 score (Supplementary Table S1).

**Figure 1.**
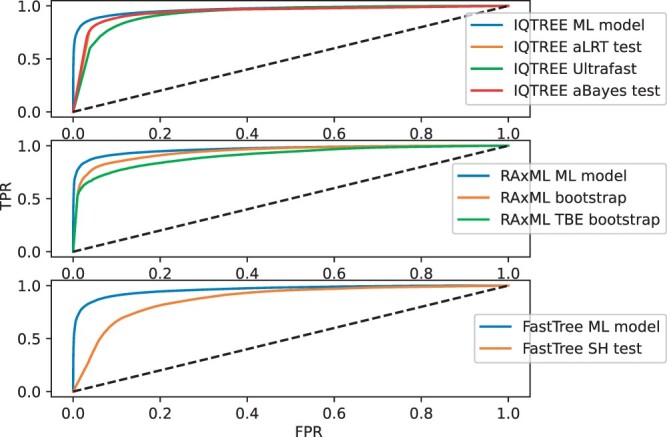
ROC curves on various test data. Each panel displays the ROC curve obtained with the branch score predictions generated using the trained machine-learning procedure compared to existing scores obtained with the respective tree search software. The top, middle, and bottom panels represent the scores obtained with trees reconstructed using IQTREE, RAxML-NG, and FastTree, respectively, on the test data. The dotted diagonal line is the *y* = *x* line. The remaining curves represent the performance of our machine-learning model along with support values provided by the other programs.

Next, we compared the performance of the machine-learning approach to that obtained by six currently used branch-support approaches, as provided by the above three tree inference software (Table 1). In all comparisons, the developed model was found to be more accurate (Fig. 1). For example, when tree searches were performed using IQTREE (Fig. 1, top panel), the machine-learning approach yielded an AUC score of 0.968 compared with ultrafast bootstrap method, which yielded an AUC score of 0.928. IQTREE also implements two additional branch-support scores, aLRT and aBayes, both of which obtained higher AUC scores than the ultrafast bootstrap, but still lower compared to the machine-learning approach (AUC scores of 0.943 and 0.942 for the aLRT test and aBayes test, respectively). The machine-learning-based branch support also demonstrated superior performance compared to the bootstrap support computed by RAxML-NG, which obtained AUC scores of 0.946 and 0.907 using either the Felsenstein’s bootstrap and the Transfer Bootstrap Expectation implementations, respectively (Fig. 1, middle panel). This analysis further revealed that, among the various scores examined, the SH test employed by FastTree exhibited the lowest performance, obtaining an AUC score of 0.876 (Fig. 1, bottom panel). Finally, we evaluated the AUC score for each MSA in the test set (DS1.b) separately, comparing the performance of our model to the other branch support approaches (see Materials and methods). Our model achieved significantly higher AUC scores compared to other branch support approaches (*P* < 10^−97^, Wilcoxon signed-rank test). These results demonstrate that the developed model exhibits superior capability in distinguishing between branches that exist in the true tree and those that do not.

Our machine-learning algorithm does not use scores obtained from any of the above three programs as features. We next examined whether further improvement can be obtained by incorporating any of the support values provided by these programs as features within the machine-learning model. However, such inclusion did not result in enhanced performance (i.e. with these features included, the same AUC scores were obtained).

### 3.2 Probabilistic interpretation of branch support values

**Figure 2.**
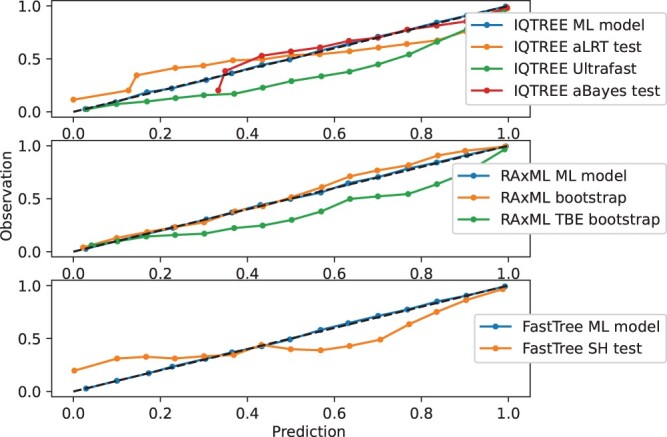
Calibration plot on the test. Each panel displays the calibration curve obtained with the branch score predictions generated using the trained machine-learning procedure compared to existing scores obtained with the respective tree search software. The top, middle, and bottom panels represent the scores obtained with trees reconstructed using IQTREE, RAxML-NG, and FastTree, respectively, on the test data. The dotted diagonal line is the *x* = *y* line. The remaining curves showcase the performance of our machine-learning model compared to other programs.

In our machine-learning model, branch-support values reflect classification probabilities, i.e. a branch support value of 70% suggests that the probability that the bipartition is found in the true tree is 70%. We next quantified how accurate these inferred probabilities are. Specifically, using simulations, we can estimate which fraction of bipartitions that were inferred to have a branch-support between, for example, 15% and 20% are found in the true tree. In a calibrated methodology, this fraction should also be between 15% and 20%. Figure 2 displays the calibration curves for all branch support methods. We quantified the calibration accuracy using the ECE and compared the machine-learning-based methodology to all alternative methods. For IQTREE, the machine-learning method demonstrated nearly perfect calibration (ECE = 0.002), i.e. almost perfect overlap with the *y *=* x* line. In contrast, the ultrafast bootstrap approach provided values much higher than the true probabilities (ECE = 0.043), i.e. it is overconfident across the entire range of support values. The aLRT obtained an ECE value almost identical to the ultrafast bootstrap (ECE = 0.04), however, it was found to be underconfident for support values below 0.5 and overconfident for support values above 0.5. The aBayes approach obtained an ECE 0.033, and was thus also inferior to that obtained by our machine-learning model. In addition, it substantially deviated from expectation for support values below 0.6 (Fig. 2, top panel). The RAxML-NG standard bootstrap values were slightly underconfident for support values above 0.5 (ECE = 0.017) (Fig. 2, middle panel). RAxML-NG Transfer Bootstrap Expectation obtained an ECE score of 0.059. Finally, FastTree branch support values substantially deviated from the expected probabilities (ECE = 0.055) (Fig. 2, bottom panel). These results indicate that when conducting tree searches with all programs, the machine-learning method demonstrated high calibration, thus providing accurate probabilistic interpretation of support values.

### 3.3 Effect of model misspecification on model performance

To assess the impact of model misspecification on the accuracy of branch-support estimates, we evaluated performance on additional validation data (see Materials and methods). In the first scenario (DS2.a), MSAs were generated without model misspecification, employing the same procedure as in the train and test datasets, to serve as a control dataset. In the second scenario (DS2.b), data were simulated using the JC model, while tree-searches were carried out assuming the GTR+F+G+I model. In the third scenario (dataset DS2.c), MSA data were simulated assuming the GTR+F+G+I model, while tree-search was performed assuming the JC model. We calculated AUC scores and generated calibration plots for DS2.b and DS2.c, comparing the results to those obtained for the control dataset DS2.a for each program. For the trained machine-learning model, under both scenarios of model misspecification, the discrimination ability of the model did not decrease (maximal decrease in AUC when compared to the control dataset is 0.001). Furthermore, our model consistently outperformed the alternative support values provided by each program (Supplementary Table S1). Regarding calibration, the control dataset (DS2.a) exhibited almost perfect calibration (ECE < 0.007 across all three programs; see Supplementary Fig. S1a), as expected. The dataset DS2.b exhibited slightly worse calibration (ECE < 0.01) (Supplementary Fig. S1b) while DS2.c resulted in poorer calibration (ECE < 0.023), particularly in IQTREE and RAxML-NG, where the model predictions showed an upward bias for support values >0.4 (Supplementary Fig. S1c). In all cases, our model was better calibrated than the other branch support scores (Supplementary Table S1).

### 3.4 Running time analysis

We compared the running times of the various branch support approaches (see Materials and methods). The computation time of RAxML-NG standard bootstrap exhibited a median running time of 138 min on a single CPU. On the same data, our machine-learning model had a median running time of 6.5 min. The most time-consuming feature in our computation is the log-likelihood evaluation of NNI neighbors. Excluding this feature had almost no effect on performance (e.g. for RAxML-NG, AUC score of 0.966 compared to 0.968 with all features), but the median running time was reduced to 7.3 s. For other programs, the branch support values are computed as part of the maximum-likelihood tree search, and hence we could not compare their running times to ours.

### 3.5 Feature analysis

Next, we analyzed which features contributed most to classification accuracy. Following a recursive feature elimination procedure (see Materials and methods), 32, 39, and 31 were selected out of 33, 39, and 33 features for the models trained for trees inferred using IQTREE, RAxML-NG, and FastTree, respectively. The features chosen by the IQTREE model are detailed in Table 2 (the importance values for all features are given in Supplementary Table S2). For all models (a model for each software), the two most important features were the minimal and maximal log-likelihood differences between the current tree and NNI trees, respectively. The next most important feature, consistently identified by all models, relies on the proportion of parsimony trees, obtained using RAxML-NG, which contain the branch of interest or its neighbors. We next tested the hypothesis that accurate predictions could be obtained by relying on a single top-scoring feature. To this end, we applied the classification algorithm with each feature separately. The most informative feature, when used alone, is the minimal log-likelihood difference between the final tree and the NNI neighbors. This feature achieves AUC scores of 0.943 in both IQTREE and RAxML-NG and 0.935 in FastTree. Although these AUC scores are high, they are lower than the AUC obtained when all features are combined (0.968, 0.968, 0.963 for the same test data in IQTREE, RAxML-NG, and FastTree, respectively). These results clearly demonstrate the need to rely on a combination of features to obtain accurate predictions.

**Table 2.** Analysis of feature importance: Gini importance for the IQTREE model and corresponding AUC values using each individual feature.

Feature name	Gini importance	AUC
Minimum log-likelihood difference between an NNI neighbor near the bipartition and current tree	861 182	0.943
Maximum log-likelihood difference between an NNI neighbor near the split and current tree	90 324	0.943
Minimum neighbor bipartition presence ratio across parsimony trees	35 207	0.734
Fraction of RAxML-NG parsimony trees in which the bipartition exists	26 831	0.915
Branch length at the partition divided by total tree divergence	21 668	0.902
Variance of branch lengths across the tree	20 365	0.593
Branch length at the partition	15 664	0.893
Mean branch length among the neighboring branches	10 983	0.73
Mean neighbor bipartition presence ratio across parsimony trees	10 676	0.734
Minimal branch length among the neighboring branches	6455	0.704
Total divergence in the smaller subtree defined by the bipartition	5482	0.582
Number of unique positions in the MSA	5291	0.644
Fraction of leaves in the smaller subtree defined by the bipartition	4632	0.521
Branch length divided by mean branch length among the neighboring branches	4462	0.904
Total divergence in the smaller subtree defined by the bipartition divided by total tree divergence	4283	0.515
Mean transfer distance of the bipartition across parsimony trees	3348	0.902
Tree MAD score	3224	0.632
MSA difficulty	3203	0.708
Number of positions in the MSA	2922	0.638
The division of the minimum branch length by the maximum branch length among the neighboring branches	2888	0.722
Skewness of tree branch lengths	2822	0.546
Number of leaves in the smaller subtree defined by the bipartition	2636	0.538
75th percentile of tree branch lengths	2448	0.656
Total tree divergence	2185	0.58
25th percentile of tree branch lengths	2030	0.692
Maximal branch length among the neighboring branches	1927	0.719
Variance of branch length among the neighboring branches	1420	0.692
Kurtosis of tree branch lengths	1344	0.532
Fraction of constant sites among the MSA sites	1155	0.56
Median of tree branch lengths	1147	0.667
Minimum of mean transfer distance from neighboring bipartitions to parsimony trees	1065	0.717
Number of sequences in the MSA	1008	0.536

### 3.6 Factors affecting model performance

We investigated various factors which might affect the performance of our machine-learning model. The model accuracy was not affected by the number of sequences (Fig 2.A), suggesting that our model is applicable across a broad spectrum of MSAs. 

**Figure 3.**
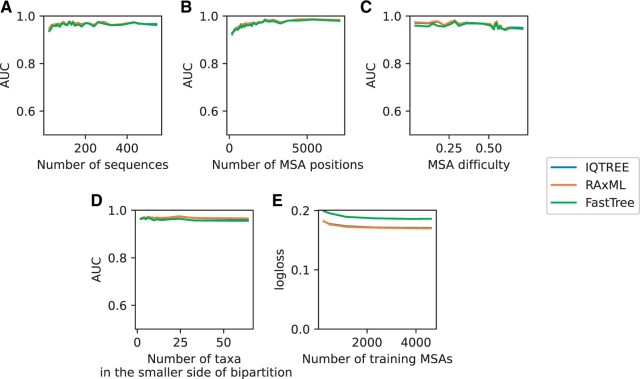
Influence of various factors on prediction accuracy in FastTree, IQTREE, and RAxML-NG models: (A) AUC as a function of the number of sequences; (B) AUC as a function of the number of MSA positions; (C) AUC as a function of MSA difficulty score; (D) AUC as a function of number of sequences in the smaller part of the bipartition (E) logarithmic loss as a function of the number of MSAs used for training. In figures A–D, the *x*-axis denotes the median value derived from dividing the numerical column into 30 quantile-based bins

As expected, the accuracy of our machine-learning model slightly increased as a function of the number of MSA positions (Fig. 3B). However, it demonstrated minimal variation with respect to the MSA difficulty score (Fig. 3C). We also tested the dependence between accuracy and the number of taxa in the smaller side of the bipartition. Here, a value of 2, for example, indicates a branch leading to a bifurcation to two species, and higher values correspond to deeper bipartitions in the tree. The accuracy was almost the same for deep versus shallow bipartitions (Fig. 3D). Finally, We evaluated whether improved performance may be obtained by increasing the size of the training data. To this end, we examined the logarithmic loss as a function of the number of MSAs used for training. Our findings indicated that accuracy reaches a plateau when the training dataset comprises 400 or more MSAs. In other words, with the current set of features, additional training data from the same source is not anticipated to yield a significant improvement in performance (Fig. 3E). In addition to the gradient boosting ensemble method (GBM), we tested the performance of the random forest model and a neural network model. Both alternative models exhibited a slight decrease in performance, with a minimum decrease of 0.002 in AUC across all software and models.

### 3.7 Applying the model on empirical MSAs

Substantial differences among branch support values were observed when analyzing 20 protein and 20 DNA empirical datasets with the various branch-support inference methodologies (see Materials and methods and Supplementary Fig. S2). The branch support is, on average, higher for our machine-learning approach compared to Felsenstein’s bootstrap and similar to that of the transfer bootstrap expectation method: the average branch support values obtained by our machine-learning model, Felsenstein’s bootstrap, and transfer bootstrap expectation were 0.85 (0.65), 0.74 (0.39), and 0.88 (0.64), for the DNA (Protein) MSAs, respectively (Supplementary Fig. S2). However, the machine-learning-based bootstrap score correlated more strongly with Felsenstein’s bootstrap than with the transfer bootstrap expectation: Pearson’s correlation coefficients (*r*) to Felsenstein’s bootstrap, and transfer bootstrap expectation were 0.73 (0.85), 0.6 (0.61), for the DNA (protein) MSAs, respectively. In comparison to parametric tests in maximum-likelihood trees obtained by IQTREE, our machine-learning approach yielded lower average support than both aLRT and aBayes support: the average support values of our machine-learning model, aLRT, and aBayes were 0.87 (0.76), 0.89 (0.84), and 0.89 (0.86), for the DNA (Protein) empirical MSAs, respectively. Both parametric tests exhibited a similar correlation with our machine-learning-based score: Pearson’s correlation coefficients to aLRT, and aBayes were 0.87 (0.79), 0.88 (0.83), for DNA (protein) MSAs, respectively.

**Figure 4.**
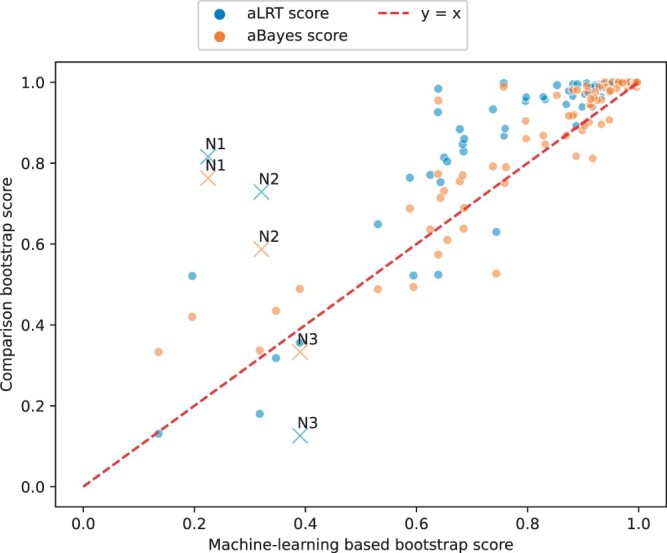
Comparison of machine-learning-based support values to aLRT and aBayes support values for the rpl16b gene using IQTREE: The *x*-axis represents the machine-learning score and the *y*-axis represents the scores of the other methods. Dots labeled as “N1” correspond to the lineage within stony corals leading to the following species: *Agaricia*, *Galaxea*, *Porites*, *Montastraea*, and *Favia*. Dots labeled as “N2” indicate support for sponge paraphyly. Dots labeled as “N3” represent the grouping of two box-jelly genera, *Carybdea* and *Tripedalia*

We next focused on the gene rpl16b from Yahalomi *et al.* (2020), which includes 701 amino-acid positions. We reconstructed the maximum-likelihood tree using IQTREE with the WAG+G model and computed three branch support values: our machine-learning approach, and the two most accurate other methods based on simulation: aBayes, and the aLRT (the last two tests are implemented in IQTREE). The correlation between the machine-learning scores and these two scores is shown in Fig. 4 (Pearson *R*^2^ of 0.84 and 0.74 between the machine-learning score and aBayes and aLRT, respectively). We searched for the nodes with the highest discrepancy between our approach and each of the two other approaches. The largest differences (for both methods) was in the lineage within stony corals leading to the following species: *Agaricia*, *Galaxea*, *Porites*, *Montastraea*, and *Favia*. For this branch, the scores for the machine-learning, aBayes, and aLRT were 0.225, 0.763, and 0.816 (see dots labeled as N1 in Fig. 4). Thus, this subclade is not supported by our methodology, while it is supported by the two others. Although we cannot determine for certain if this clade is indeed incorrect, we note that it disagrees with the tree reconstructed by the entire set of 78 protein-coding genes given in Yahalomi *et al.* (2020). Another large discrepancy concerns the sponge monophyly. Sponges were paraphyletic in the maximum-likelihood tree of this protein, because hexactinellid sponges were grouped together with ctenophores, placozoans, and cnidarians (rather than with the other sponges). The support for this grouping was above 0.5 for aBayes and aLRT (0.587 and 0.729, respectively. See dots labeled as N2 in Fig. 4). In contrast, the support for the machine-learning methodology was 0.32. Of note, most current research, and the tree based on the entire set of genes support sponge monophyly (Pick *et al.* 2010, Yahalomi *et al.* 2020). Our method often provides lower support compared to aBayes and aLRT (average support values over all nodes of 0.87, 0.9, 0.92 for the machine-learning approach, aBayes, and aLRT, respectively). However, in a few cases, our approach provided higher support compared to the two other ones, e.g. for the grouping of two box-jelly genera, *Carybdea* and *Tripedalia*, the three support values were 0.39,0.33,0.13 for the machine-learning approach, aBayes, and aLRT, respectively (see dots labeled as N3 in Fig. 4). Of note, this node is supported when information from all 78 proteins are considered (Yahalomi *et al.* 2020).

## 4 Discussion

Recently, machine-learning algorithms were successfully applied in phylogenetic research, contributing to both runtime efficiency and enhanced inference accuracy. Noteworthy applications include their utilization in model selection tasks (Abadi *et al.* 2020, Burgstaller-Muehlbacher *et al.* 2023), inferring phylogenetic trees (Suvorov *et al.* 2020), ranking candidate trees during a tree-search (Azouri *et al.* 2021), identification of key genomic loci for elucidating a phylogenetic hypothesis (Kumar and Sharma 2021), sampling of MSA positions to reduce tree-search running time (Ecker *et al.* 2022), and estimating the difficulty of the MSA (Haag *et al.* 2022). In this study, we have demonstrated the effectiveness of machine-learning algorithms for branch support estimation, a task traditionally relying on standard statistical tests. We developed a machine-learning classification model to estimate branch support for phylogenies reconstructed using a variety of maximum-likelihood search algorithms. The model was trained using thousands of MSAs which were simulated based on realistic phylogenetic trees, assuming various DNA models. We demonstrated that our methodology provides precise and fast branch support estimates for maximum-likelihood trees obtained using state-of-the-art tree-search software. Furthermore, the developed machine-learning approach outperformed common branch support methodologies in terms of its probabilistic interpretation. We have also shown that our classifier remains accurate under model misspecification scenarios. Finally, the empirical analysis suggests that substantial differences may be obtained by employing different branch-support methodologies, and together with the simulation results, suggest that this machine-learning methodology provides reliable estimate of branch support and should be incorporated in standard phylogenetic software.

The features incorporated into this model encompass log-likelihood evaluation, including branch-length optimization, for the three NNI neighbors of each bipartition. It is worth noting that these computations or their approximations are typically executed during a tree-search, incurring no additional computational cost. Nevertheless, even when these features are removed, the model still produced favorable results (maximum difference in AUC compared to the original model across the three programs is 0.005; see Supplementary Table S1).

In the development of our machine-learning models, we employed hand-crafted features specifically designed for estimating branch support. While these features exhibit strong predictive power, further improvement can be potentially achieved by adopting a more comprehensive numerical representation of the maximum-likelihood tree and MSA. The MSA can be represented numerically using an unsupervised learning model, such as the one employed by Facebook’s protein language model (Rao *et al.* 2021), while the nodes within the maximum-likelihood tree can be embedded in high-dimensional space using graph-based embeddings techniques (Cai *et al.* 2018, Matsumoto *et al.* 2021). Such an approach holds the potential to capture complex and relevant characteristics more effectively.

In all analyses performed here, it was assumed that the MSA is correct. However, the MSA is inferred, and alignment errors were shown to impact many downstream analyses, including tree topology search and bootstrap estimates (e.g. Wong *et al.* 2008). Ideally, uncertainty in the MSA should be accounted for within the estimate of branch support. This can be achieved within Bayesian approaches, which jointly infer the posterior distributions of alignments and trees (Redelings 2021). However, how to integrate alignment uncertainty within a frequentist inference framework is more challenging. It is possible to repeat the tree search and the branch-support inference for a set of alternative alignments and assign each branch the average support over these alternative alignments (Chatzou *et al.* 2018, Chang *et al.* 2021). A set of alternative alignments can be generated by running different alignments programs, by considering co-optimal alignment solutions (Landan and Graur 2007), or by integrating uncertainty in gap scoring, guide tree, and co-optimal solutions together (Sela *et al.* 2015).

Our analysis further tested the sensitivity of the developed branch-support estimates to model misspecification. The type of misspecification we evaluated focused on the transition rates between nucleotide substitutions. In many cases, the continuous-time Markov models that are currently included in tree inference software may be oversimplified. For example, all models assume that within each site, the evolutionary rate is constant. However, when divergent sequences are analyzed, it is often the case that a site is conserved in one part of the tree and variable in the rest of tree or vice versa (Pupko and Galtier 2002, Wang *et al.* 2007). Similarly, it is assumed that the models are stationary and reversible, which may not be the case (e.g. Barba-Montoya *et al.* 2020). For example, when bacterial sequences are analyzed, assuming the same GC content along the tree was shown to lead to tree reconstruction artifacts (Galtier and Gouy 1998). The sensitivity of branch-support metrics to these and other model misspecifications deserves further research.

The standard Felsenstein’s bootstrap as well as the other branch support estimates studied in this work, all assume that sequence sites evolve independently of one another. This is clearly not the case for most biological sequences, e.g. conserved columns within an MSA tend to cluster together, pointing to some functionally important 3D regions (Guharoy and Chakrabarti 2010). Several previous efforts aimed to explicitly model sequence data, aiming to alleviate the assumption of site independence by incorporating probabilistic Markov processes (Von Haeseler and Schöniger 1998, Lunter and Hein 2004, Larson *et al.* 2020, Chang *et al.* 2021). However, these models are seldomly used in tree-search algorithms. In theory, methods such as the block bootstrap can be used to account for this nonindependence (Kunsch 1989). Of note, not accounting for such nonindependence is likely to highly inflate branch-support errors (Holmes 2003). The machine-learning approach can be adapted to account for dependence of the evolutionary process among sites. This necessitates simulating a large number of datasets along a known tree, assuming site dependence. Unfortunately, current software for simulating sequences along a tree does not enable simulations with site dependence.

## Supplementary Material

btae255_Supplementary_Data
