# Supplementary material for: A machine-learning-based alternative to phylogenetic bootstrap
Source: Bioinformatics. 2024 Jun 28;40(Suppl 1):i208–17. doi: 10.1093/bioinformatics/btae255 (PMC11211842; doi:10.1093/bioinformatics/btae255)
Supplement: btae255_Supplementary_Data [file btae255_supplementary_data.pdf]

Supplementary information

A machine-learning-based alternative to phylogenetic bootstrap

Noa Ecker[1], Dorothée Huchon[2,3], Yishay Mansour[4], Itay Mayrose[5], Tal Pupko[1†]

[1] The Shmunis School of Biomedicine and Cancer Research, George S. Wise Faculty of Life Sciences, Tel Aviv University, Tel Aviv 6997801, Israel. [2] School of Zoology, George S. Wise Faculty of Life Sciences, Tel Aviv University, Tel Aviv 6997801, Israel. [3]The Steinhardt Museum of Natural History and National Research Center, Tel Aviv University, Tel Aviv, 6997801 Israel [4]The Blavatnik School of Computer Science, Raymond & Beverly Sackler Faculty of Exact Sciences, Tel Aviv University, Tel Aviv 6997801, Israel. [5] School of Plant Sciences and Food Security, George S. Wise Faculty of Life Sciences, Tel Aviv University, Tel Aviv 6997801, Israel.

[†]To whom correspondence should be addressed.

Associate Editor: XXXXXXX

Received on XXXXX; revised on XXXXX; accepted on XXXXX

Tables

Table S1.

Performance of classification model on training-data (DS1.a), test-data (DS1.b) and validation-data (DS2) for RAxML-NG (A), FastTree (B) and IQTREE (C)

A.

Features	Dataset	AUC(*)	ECE(**)	MCC (***)	FPR (****)	FNR (****)	F1 score
All features	Test	0.968	0.002	0.735	0.054	0.192	0.781
All features	Train	0.974	0.006	0.759	0.049	0.174	0.801
All features	Validation-GTR+F+I+G -> JC	0.965	0.018	0.718	0.041	0.246	0.761
All features	Validation-control	0.966	0.005	0.716	0.048	0.232	0.763
All features	Validation-JC -> GTR+F+I+G	0.973	0.006	0.766	0.044	0.165	0.802
All features except NNI features	Test	0.965	0.002	0.727	0.05	0.214	0.774
All features except NNI features	Train	0.972	0.006	0.749	0.047	0.196	0.793
All features except NNI features	Validation-GTR+F+I+G -> JC	0.961	0.011	0.717	0.041	0.249	0.76
All features except NNI features	Validation-control	0.963	0.004	0.709	0.045	0.254	0.756
All features except NNI features	Validation-JC -> GTR+F+I+G	0.97	0.006	0.758	0.043	0.18	0.795
Raw standard bootstrap support	Test	0.944	0.017	0.649	0.043	0.347	0.702
Raw standard bootstrap support	Train	0.946	0.017	0.658	0.04	0.344	0.71
Raw standard bootstrap support	Validation-GTR+F+I+G -> JC	0.946	0.019	0.668	0.042	0.314	0.716
Raw standard bootstrap support	Validation-control	0.942	0.016	0.637	0.04	0.367	0.688
Raw standard bootstrap support	Validation-JC -> GTR+F+I+G	0.956	0.018	0.71	0.035	0.28	0.751
Single feature standard bootstrap support	Test	0.944	0.003	0.649	0.043	0.347	0.702
Single feature standard bootstrap support	Train	0.946	0	0.658	0.04	0.344	0.71

Single feature standard bootstrap support	Validation-GTR+F+I+G -> JC	0.946	0.009	0.668	0.042	0.314	0.716
Single feature standard bootstrap support	Validation-control	0.942	0.005	0.637	0.04	0.367	0.688
Single feature standard bootstrap support	Validation-JC -> GTR+F+I+G	0.956	0.009	0.71	0.035	0.28	0.751
All features + standard bootstrap support	Test	0.968	0.001	0.739	0.054	0.187	0.784
All features + standard bootstrap support	Train	0.974	0.005	0.759	0.05	0.172	0.802
All features + standard bootstrap support	Validation-GTR+F+I+G -> JC	0.968	0.015	0.727	0.042	0.232	0.769
All features + standard bootstrap support	Validation-control	0.967	0.004	0.718	0.049	0.228	0.765
All features + standard bootstrap support	Validation-JC -> GTR+F+I+G	0.975	0.005	0.769	0.044	0.16	0.805
Raw TBE standard bootstrap support	Test	0.907	0.059	0.403	0.012	0.748	0.385
Raw TBE standard bootstrap support	Train	0.91	0.06	0.411	0.011	0.743	0.391
Raw TBE standard bootstrap support	Validation-GTR+F+I+G -> JC	0.912	0.051	0.413	0.013	0.73	0.401
Raw TBE standard bootstrap support	Validation-control	0.907	0.056	0.406	0.011	0.75	0.383
Raw TBE standard bootstrap support	Validation-JC -> GTR+F+I+G	0.926	0.046	0.456	0.011	0.701	0.44
Single feature TBE standard bootstrap support	Test	0.908	0.003	0.477	0.035	0.584	0.524
Single feature TBE standard bootstrap support	Train	0.912	0.001	0.486	0.033	0.582	0.531
Single feature TBE standard bootstrap support	Validation-GTR+F+I+G -> JC	0.913	0.007	0.485	0.033	0.575	0.528
Single feature TBE standard bootstrap support	Validation-control	0.908	0.008	0.466	0.036	0.593	0.512
Single feature TBE standard bootstrap support	Validation-JC -> GTR+F+I+G	0.928	0.011	0.543	0.027	0.532	0.579
All features + standard TBE bootstrap support	Test	0.968	0.002	0.738	0.053	0.19	0.783

All features + standard TBE bootstrap support	Train	0.974	0.005	0.758	0.049	0.175	0.801
All features + standard TBE bootstrap support	Validation-GTR+F+I+G -> JC	0.967	0.016	0.724	0.041	0.238	0.766
All features + standard TBE bootstrap support	Validation-control	0.967	0.006	0.72	0.048	0.227	0.766
All features + standard TBE bootstrap support	Validation-JC -> GTR+F+I+G	0.975	0.004	0.765	0.044	0.165	0.802

B.

Features	Dataset	AUC(*)	ECE(**)	MCC (***)	FPR (****)	FNR (****)	F1 score
All features	Test	0.963	0.002	0.732	0.056	0.197	0.782
All features	Train	0.972	0.006	0.753	0.051	0.182	0.799
All features	Validation-GTR+F+I+G -> JC	0.962	0.014	0.71	0.048	0.233	0.755
All features	Validation-control	0.957	0.007	0.707	0.051	0.244	0.759
All features	Validation-JC -> GTR+F+I+G	0.974	0.007	0.763	0.044	0.171	0.8
All features except NNI features	Test	0.957	0.003	0.706	0.052	0.245	0.759
All features except NNI features	Train	0.967	0.007	0.731	0.048	0.225	0.78
All features except NNI features	Validation-GTR+F+I+G -> JC	0.962	0.006	0.708	0.048	0.235	0.754
All features except NNI features	Validation-control	0.95	0.006	0.675	0.047	0.303	0.729
All features except NNI features	Validation-JC -> GTR+F+I+G	0.971	0.013	0.747	0.045	0.189	0.786
Raw SH support	Test	0.876	0.055	0.506	0.034	0.557	0.555
Raw SH support	Train	0.875	0.058	0.498	0.033	0.571	0.544
Raw SH support	Validation-GTR+F+I+G -> JC	0.842	0.058	0.39	0.022	0.717	0.403
Raw SH support	Validation-control	0.87	0.057	0.469	0.031	0.609	0.509
Raw SH support	Validation-JC -> GTR+F+I+G	0.885	0.041	0.521	0.029	0.551	0.559

Single feature SH support	Test	0.876	0.004	0.551	0.057	0.434	0.622
Single feature SH support	Train	0.876	0.001	0.548	0.054	0.446	0.617
Single feature SH support	Validation-GTR+F+I+G -> JC	0.841	0.017	0.457	0.041	0.579	0.51
Single feature SH support	Validation-control	0.871	0.01	0.515	0.057	0.476	0.587
Single feature SH support	Validation-JC -> GTR+F+I+G	0.886	0.017	0.599	0.044	0.398	0.653
All features + SH support	Test	0.963	0.002	0.732	0.056	0.197	0.782
All features + SH support	Train	0.97	0.006	0.753	0.051	0.182	0.799
All features + SH support	Validation-GTR+F+I+G -> JC	0.962	0.013	0.713	0.047	0.231	0.757
All features + SH support	Validation-control	0.957	0.006	0.707	0.051	0.244	0.758
All features + SH support	Validation-JC -> GTR+F+I+G	0.973	0.006	0.758	0.044	0.177	0.796

C.

Features	Dataset	AUC(*)	ECE**)	MCC (***)	FPR (****)	FNR (****)	F1 score
All features	Test	0.968	0.002	0.737	0.053	0.193	0.783
All features	Train	0.974	0.006	0.758	0.049	0.176	0.801
All features	Validation-GTR+F+I+G -> JC	0.962	0.023	0.698	0.042	0.271	0.743
All features	Validation-control	0.966	0.006	0.712	0.048	0.24	0.76
All features	Validation-JC -> GTR+F+I+G	0.972	0.01	0.748	0.043	0.195	0.786
All features except NNI features	Test	0.964	0.002	0.725	0.05	0.22	0.773
All features except NNI features	Train	0.972	0.006	0.747	0.046	0.201	0.791
All features except NNI features	Validation-GTR+F+I+G -> JC	0.958	0.015	0.697	0.041	0.278	0.741
All features except NNI features	Validation-control	0.963	0.006	0.696	0.045	0.272	0.745
All features except NNI features	Validation-JC -> GTR+F+I+G	0.969	0.007	0.742	0.042	0.209	0.781
Raw ultrafast bootstrap support	Test	0.928	0.043	0.572	0.013	0.561	0.584

Raw ultrafast bootstrap support	Train	0.929	0.042	0.583	0.013	0.547	0.597
Raw ultrafast bootstrap support	Validation-GTR+F+I+G -> JC	0.933	0.037	0.565	0.013	0.569	0.574
Raw ultrafast bootstrap support	Validation-control	0.93	0.038	0.566	0.012	0.573	0.574
Raw ultrafast bootstrap support	Validation-JC -> GTR+F+I+G	0.946	0.032	0.595	0.011	0.541	0.603
Single feature ultrafast bootstrap support	Test	0.928	0.003	0.663	0.044	0.326	0.716
Single feature ultrafast bootstrap support	Train	0.929	0	0.667	0.043	0.321	0.72
Single feature ultrafast bootstrap support	Validation-GTR+F+I+G -> JC	0.933	0.009	0.638	0.041	0.356	0.687
Single feature ultrafast bootstrap support	Validation-control	0.93	0.008	0.654	0.045	0.33	0.707
Single feature ultrafast bootstrap support	Validation-JC -> GTR+F+I+G	0.946	0.013	0.677	0.035	0.326	0.721
All features+ ultrafast bootstrap support	Test	0.968	0.002	0.737	0.052	0.195	0.783
All features+ ultrafast bootstrap support	Train	0.974	0.005	0.759	0.048	0.178	0.801
All features+ ultrafast bootstrap support	Validation-GTR+F+I+G -> JC	0.964	0.019	0.709	0.043	0.253	0.753
All features+ ultrafast bootstrap support	Validation-control	0.967	0.005	0.715	0.047	0.238	0.762
All features+ ultrafast bootstrap support	Validation-JC -> GTR+F+I+G	0.974	0.005	0.762	0.043	0.174	0.799
Raw aLRT support	Test	0.943	0.04	0.706	0.051	0.242	0.757
Raw aLRT support	Train	0.944	0.039	0.713	0.05	0.236	0.763
Raw aLRT support	Validation-GTR+F+I+G -> JC	0.938	0.047	0.694	0.044	0.269	0.74
Raw aLRT support	Validation-control	0.941	0.043	0.681	0.049	0.278	0.733
Raw aLRT support	Validation-JC -> GTR+F+I+G	0.937	0.038	0.733	0.044	0.21	0.774
Single feature aLRT support	Test	0.943	0.002	0.708	0.053	0.233	0.758
Single feature aLRT support	Train	0.945	0	0.716	0.052	0.226	0.766

Single feature aLRT support	Validation-GTR+F+I+G -> JC	0.938	0.02	0.696	0.045	0.264	0.741
Single feature aLRT support	Validation-control	0.941	0.007	0.686	0.051	0.265	0.738
Single feature aLRT support	Validation-JC -> GTR+F+I+G	0.936	0.013	0.733	0.046	0.206	0.774
All features+ aLRT support	Test	0.968	0.002	0.735	0.053	0.196	0.781
All features+ aLRT support	Train	0.973	0.006	0.757	0.049	0.179	0.8
All features+ aLRT support	Validation-GTR+F+I+G -> JC	0.962	0.023	0.699	0.042	0.269	0.744
All features+ aLRT support	Validation-control	0.965	0.005	0.708	0.048	0.245	0.756
All features+ aLRT support	Validation-JC -> GTR+F+I+G	0.972	0.01	0.746	0.042	0.2	0.784
Raw aBayes support	Test	0.942	0.031	0.709	0.059	0.211	0.76
Raw aBayes support	Train	0.944	0.032	0.717	0.058	0.205	0.767
Raw aBayes support	Validation-GTR+F+I+G -> JC	0.94	0.033	0.698	0.045	0.259	0.744
Raw aBayes support	Validation-control	0.941	0.03	0.69	0.057	0.239	0.742
Raw aBayes support	Validation-JC -> GTR+F+I+G	0.938	0.03	0.736	0.048	0.192	0.776
Single feature aaBayes support	Test	0.942	0.003	0.703	0.051	0.247	0.754
Single feature aaBayes support	Train	0.944	0	0.712	0.049	0.24	0.762
Single feature aaBayes support	Validation-GTR+F+I+G -> JC	0.94	0.017	0.69	0.041	0.285	0.735
Single feature aaBayes support	Validation-control	0.94	0.005	0.68	0.048	0.285	0.731
Single feature aaBayes support	Validation-JC -> GTR+F+I+G	0.938	0.008	0.731	0.044	0.216	0.772
All features+ aBayes support	Test	0.968	0.002	0.735	0.053	0.196	0.782
All features+ aBayes support	Train	0.973	0.006	0.757	0.049	0.179	0.8

(*) Area Under the ROC curve
(**) Expected calibration Error
(***) Matthews correlation coefficient
(****) False Positive Rate, False Negative Rate

Table S2.

Analysis of feature importance: Gini Importance and corresponding AUC values using each individual Feature for the (A) RAxML model and (B) FastTree model

A.

Feature name	Gini importance	AUC
Minimum log-likelihood difference between an NNI neighbour near the bipartition and current tree	896,971	0.943
Maximum log-likelihood difference between an NNI neighbour near the split and current tree	45,227	0.942
Fraction of parsimoy trees in which the bipartition exists	28,009	0.915
Branch length at the parititon divided by total tree divergence	25,116	0.903
Mean Neighbour Bipartition Presence Ratio across final ML trees	21,454	0.715
Variance of branch lengths across the tree	16,421	0.582
Minimum neighbour Bipartition Presence Ratio across parsimony trees	16,141	0.734
Mean Transfer Distance of the bipartition across final ML trees	14,987	0.759
Fraction of final ML trees in which the bipartition exists	7,098	0.76
Minimum Neighbour Bipartition Presence Ratio across final ML trees	6,913	0.715
Branch length at the parititon	5,655	0.891
Mean neighbour Bipartition Presence Ratio across parsimony trees	5,227	0.735
Mean branch length among the neighbouring branches	5,197	0.728
Total divergence in the smaller subtree defined by the bipartition	4,988	0.581
Mean Neighbour Bipartition Presence Ratio across final ML trees	4,961	0.714
Minimal branch length among the neighbouring branches	4,718	0.696
Number of unique positions in the MSA	4,505	0.644
Branch length divided by mean branch length among the neighbouring branches	4,230	0.919
Fraction of leaves in the smaller subtree defined by the bipartition	4,185	0.52

Total divergence in the smaller subtree defined by the bipartition divided by total tree divergence	3,658	0.51
Tree MAD score	3,074	0.632
Minimum of mean Transfer Distance from Neighbouring Bipartitions to final ML trees Trees	3,069	0.713
Mean Transfer Distance of the bipartition across parsimoy trees	2,803	0.902
Number of leaves in the smaller subtree defined by the bipartition	2,403	0.538
Skewness of tree branch lengths	2,399	0.544
75th percentile of tree branch lengths	2,217	0.652
Number of positions in the MSA	2,112	0.637
The division of the minimum branch length by the maximum branch length among the neighbouring branches	1,811	0.729
MSA difficulty	1,758	0.705
25th percentile of tree branch lengths	1,675	0.683
Number of sequences in the MSA	1,598	0.535
Maximal branch length among the neighbouring branches	1,561	0.717
Variance of branch length among the neighbouring branches	1,411	0.689
Fraction of constant sites among the MSA sites	1,399	0.558
Total tree divergence	1,266	0.583
Median of tree branch lengths	1,252	0.662
Kurtosis of tree branch lengths	986	0.526
Minimum of mean Transfer Distance from Neighbouring Bipartitions to parsimony Trees	804	0.718
Overall mean of Transfer Distance from Neighbouring Bipartitions to parsimony Trees	781	0.723

B.

Feature name	Gini importance	AUC
Minimum log-likelihood difference between an NNI neighbour near the bipartition and current tree	859,683	0.935
Maximum log-likelihood difference between an NNI neighbour near the split and current tree	100,558	0.934
Fraction of parsimoy trees in which the bipartition exists	51,010	0.916
Minimum neighbour Bipartition Presence Ratio across parsimony trees	30,679	0.74
Mean neighbour Bipartition Presence Ratio across parsimony trees	17,069	0.739
Skewness of tree branch lengths	13,208	0.572
Variance of branch lengths across the tree	12,242	0.576
Mean branch length among the neighbouring branches	7,646	0.712
Branch length at the parititon divided by total tree divergence	7,539	0.804
Total divergence in the smaller subtree defined by the bipartition	7,310	0.571
Minimal branch length among the neighbouring branches	5,469	0.688
Mean Transfer Distance of the bipartition across parsimoy trees	5,202	0.902
Total divergence in the smaller subtree defined by the bipartition divided by total tree divergence	5,119	0.518
Branch length at the parititon	5,021	0.865
Fraction of leaves in the smaller subtree defined by the bipartition	4,513	0.527
Tree MAD score	3,860	0.608
Branch length divided by mean branch length among the neighbouring branches	3,560	0.798
MSA difficulty	3,356	0.702
Fraction of constant sites among the MSA sites	3,029	0.556
Number of sequences in the MSA	2,932	0.532
Number of leaves in the smaller subtree defined by the bipartition	2,912	0.541
Total tree divergence	2,792	0.564

75th percentile of tree branch lengths	2,788	0.646
Number of unique positions in the MSA	2,730	0.643
Number of positions in the MSA	2,294	0.638
Overall mean of Transfer Distance from Neighbouring Bipartitions to parsimony Trees	2,233	0.73
Maximal branch length among the neighbouring branches	2,215	0.703
Variance of branch length among the neighbouring branches	2,119	0.68
Minimum of mean Transfer Distance from Neighbouring Bipartitions to parsimony Trees	1,460	0.725
The division of the minimum branch length by the maximum branch length among the neighbouring branches	1,446	0.548
25th percentile of tree branch lengths	1,325	0.665

Table S3.

Publications corresponding to empirical datasets from Rob Lanfear's collection used for external validation of the machine-learning model.

Study name	Study reference
Wainwright_2012	Wainwright PC, Smith WL, Price SA, Tang KL, Sparks JS, Ferry LA, Kuhn KL, Eytan RI, Near TJ. The evolution of pharyngognathy: a phylogenetic and functional appraisal of the pharyngeal jaw key innovation in labroid fishes and beyond. Systematic Biology. 2012 Dec 1;61(6):1001-27.
Seago_2011	Seago AE, Giorgi JA, Li J, Ślipiński A. Phylogeny, classification and evolution of ladybird beetles (Coleoptera: Coccinellidae) based on simultaneous analysis of molecular and morphological data. Molecular Phylogenetics and Evolution. 2011 Jul 31;60(1):137-51.
Murray_2013	Murray EA, Carmichael AE, Heraty JM. Ancient host shifts followed by host conservatism in a group of ant parasitoids. Proceedings of the Royal Society of London B: Biological Sciences. 2013 May 22;280(1759):20130495.
Unmack_2013	Unmack PJ, Allen GR, Johnson JB. Phylogeny and biogeography of rainbowfishes (Melanotaeniidae) from Australia and New Guinea. Molecular Phylogenetics and Evolution. 2013 Apr 30;67(1):15-27.
Wood_2012	Wood HM, Matzke NJ, Gillespie RG, Griswold CE. Treating fossils as terminal taxa in divergence time estimation reveals ancient vicariance patterns in the palpimanoid spiders. Systematic Biology. 2012 Nov 28:sys092.
Day_2013	Day JJ, Peart CR, Brown KJ, Friel JP, Bills R, Moritz T. Continental diversification of an African catfish radiation (Mochokidae: Synodontis). Systematic biology. 2013 Jan 9:syt001.
Kawahara_2013	Kawahara AY, Rubinoff D. Convergent evolution of morphology and habitat use in the explosive Hawaiian fancy case caterpillar radiation. Journal of evolutionary biology. 2013 Aug 1;26(8):1763-73.

Bergsten_2013	Bergsten J, Nilsson AN, Ronquist F. Bayesian tests of topology hypotheses with an example from diving beetles. Systematic biology. 2013 Sep 1;62(5):660-73.
Worobey_2014h	Worobey M, Han G, Rambaut A (2014) A synchronized global sweep of the internal genes of modern avian influenza virus. Nature 508(7495): 254,Äì257.
Worobey_2014a	Worobey M, Han G, Rambaut A (2014) A synchronized global sweep of the internal genes of modern avian influenza virus. Nature 508(7495): 254,Äì257.
Worobey_2014f	Worobey M, Han G, Rambaut A (2014) A synchronized global sweep of the internal genes of modern avian influenza virus. Nature 508(7495): 254,Äì257.
Siler_2013	Siler CD, Oliveros CH, Santanen A, Brown RM. Multilocus phylogeny reveals unexpected diversification patterns in Asian wolf snakes (genus Lycodon). Zoologica Scripta. 2013 May 1;42(3):262-77.
Cognato_2001	Cognato AI, Vogler AP. Exploring data interaction and nucleotide alignment in a multiple gene analysis of Ips (Coleoptera: Scolytinae). Systematic Biology. 2001 Nov 1;50(6):758-80.
Worobey_2014g	Worobey M, Han G, Rambaut A (2014) A synchronized global sweep of the internal genes of modern avian influenza virus. Nature 508(7495): 254,Äì257.
Brown_2012	Brown RM, Siler CD, Das I, Min Y. Testing the phylogenetic affinities of Southeast Asia,Äôs rarest geckos: Flap-legged geckos (Luperosaurus), Flying geckos (Ptychozoon) and their relationship to the pan-Asian genus Gekko. Molecular phylogenetics and evolution. 2012 Jun 30;63(3):915-21.
Sauquet_2011	Sauquet H, Ho SY, Gandolfo MA, Jordan GJ, Wilf P, Cantrill DJ, Bayly MJ, Bromham L, Brown GK, Carpenter RJ, Lee DM. Testing the impact of calibration on molecular divergence times using a fossil-rich group: the case of Nothofagus (Fagales). Systematic Biology. 2012 Mar 1;61(2):289-313.
Anderson_2013	Anderson FE, Bergman A, Cheng SH, Pankey MS, Valinassab T. Lights out: the evolution of bacterial bioluminescence in Loliginidae. Hydrobiologia. 2014 Mar 1;725(1):189-203.
Devitt_2013	Devitt TJ, Devitt SE, Hollingsworth BD, McGuire JA, Moritz C. Montane refugia predict population genetic structure in the Large,Äêblotched Ensatina salamander. Molecular ecology. 2013 Mar 1;22(6):1650-65.
Oaks_2011	Oaks JR. A time-calibrated species tree of Crocodylia reveals a recent radiation of the true crocodiles. Evolution. 2011 Nov 1;65(11):3285-97.
Dornburg_2012	Dornburg A, Moore JA, Webster R, Warren DL, Brandley MC, Iglesias TL, Wainwright PC, Near TJ. Molecular phylogenetics of squirrelfishes and soldierfishes (Teleostei: Beryciformes: Holocentridae): Reconciling more than 100 years of taxonomic confusion. Molecular phylogenetics and Evolution. 2012 Nov 30;65(2):727-38.

Figures

Fig S1.

A.

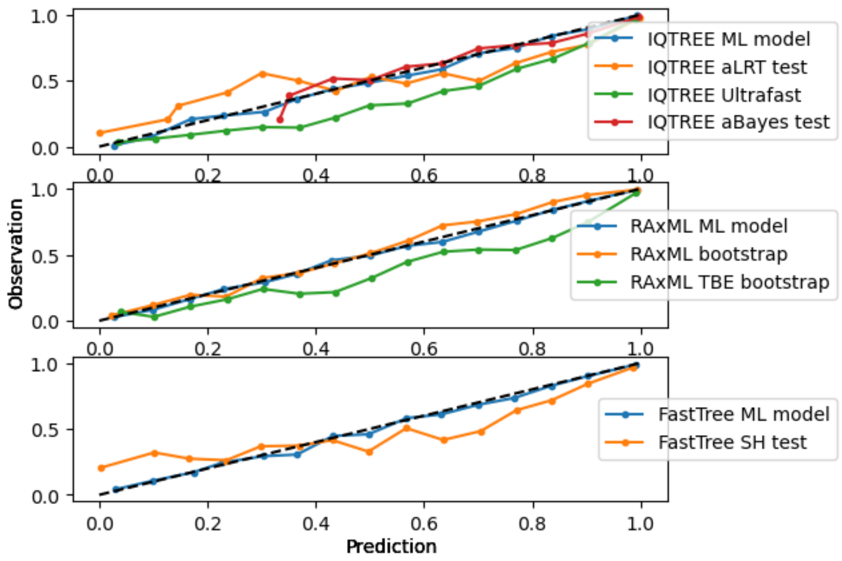

Calibration plot on the control validation data for IQTREE, RAxML-NG and FastTree. The black dotted line is the x=y line. The blue line in each figure represents the performance of our machine-learning model, while the remaining lines showcase the performance of other support values provided by the program.

B.

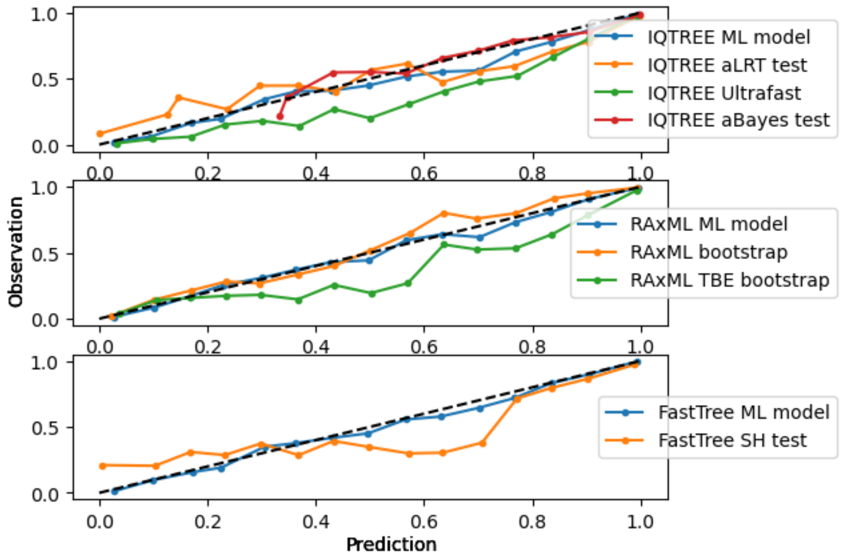

Calibration plot on the JTT->GTR+F+I+G validation dataset for IQTREE, RAxML-NG and FastTree. The black dotted line is the x=y line. The blue line in each figure represents the performance of our machine-

learning model, while the remaining lines showcase the performance of other support values provided by the program.

C.

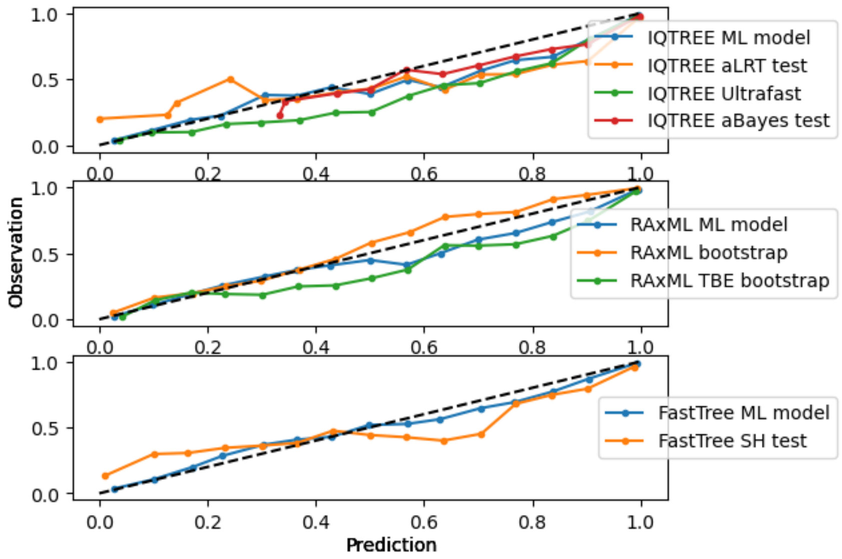

Calibration plot on the GTR+F+I+G->JTT validation dataset for IQTREE, RAxML-NG and FastTree. The black dotted line is the x=y line. The blue line in each figure represents the performance of our machine-learning model, while the remaining lines showcase the performance of other support values provided by the program.

Fig S2.

A.

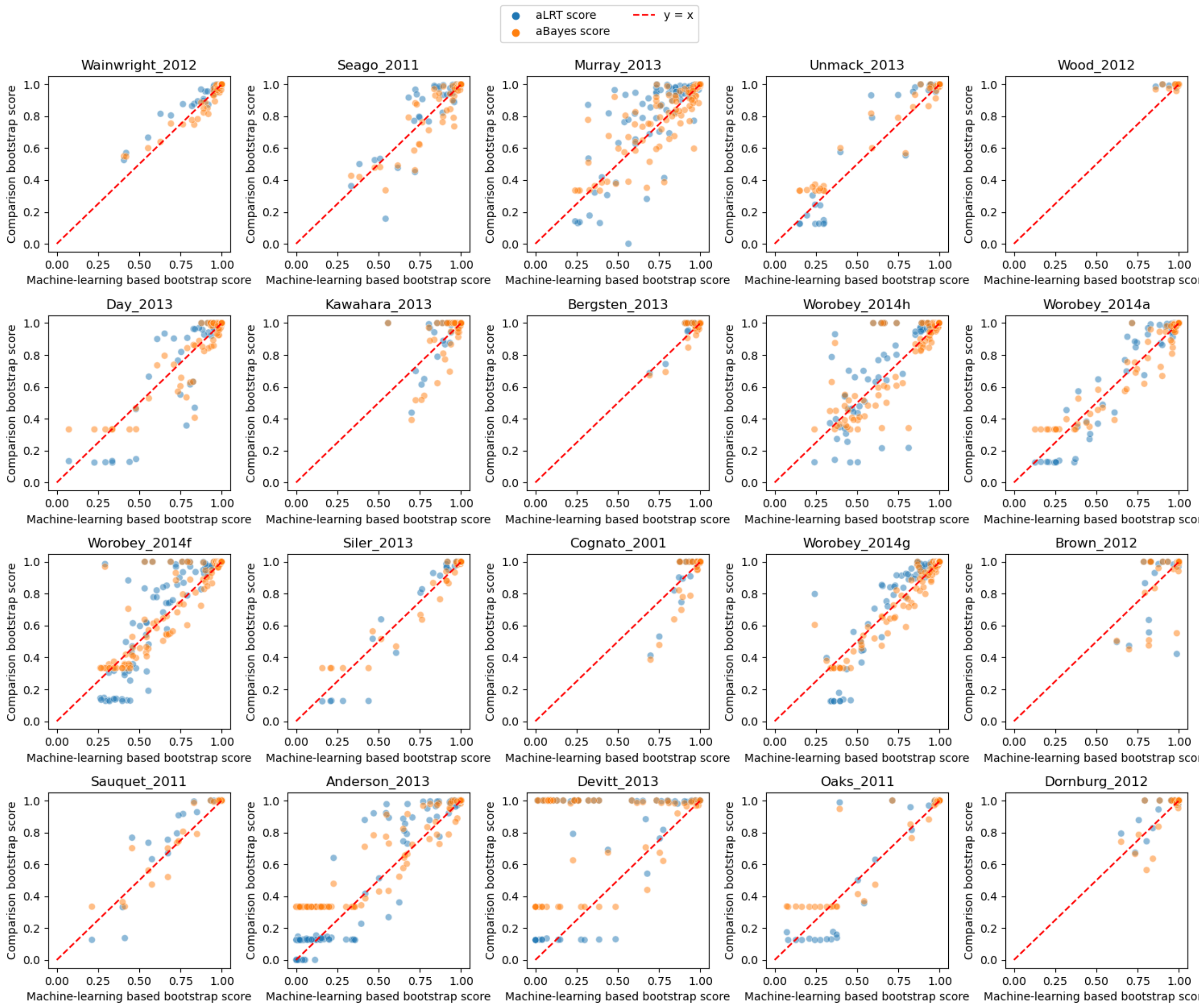

Comparison of the machine-learning bootstrap prediction with aBayes score and aLRT score implemented in IQTREE. Comparison is performed on 20 empirical DNA datasets from Rob Lanfear's The red dashed line represents the diagonal x=y line.

B.

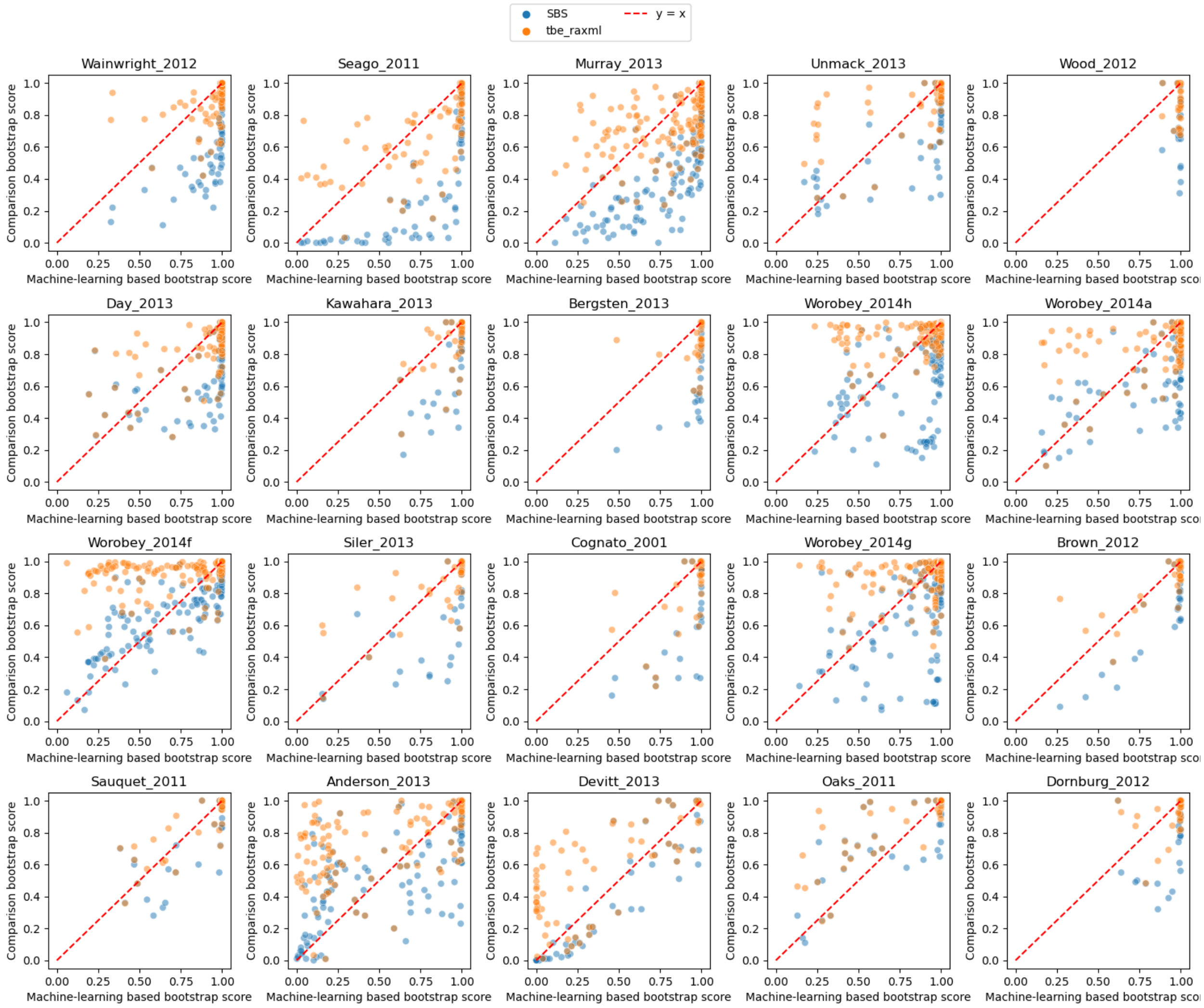

Comparison of the machine-learning bootstrap prediction with the standard bootstrap score and TBE bootstrap score implemented in RAxML-NG. Comparison is performed on 20 empirical DNA datasets from Rob Lanfear's The red dashed line represents the diagonal x=y line.

C.

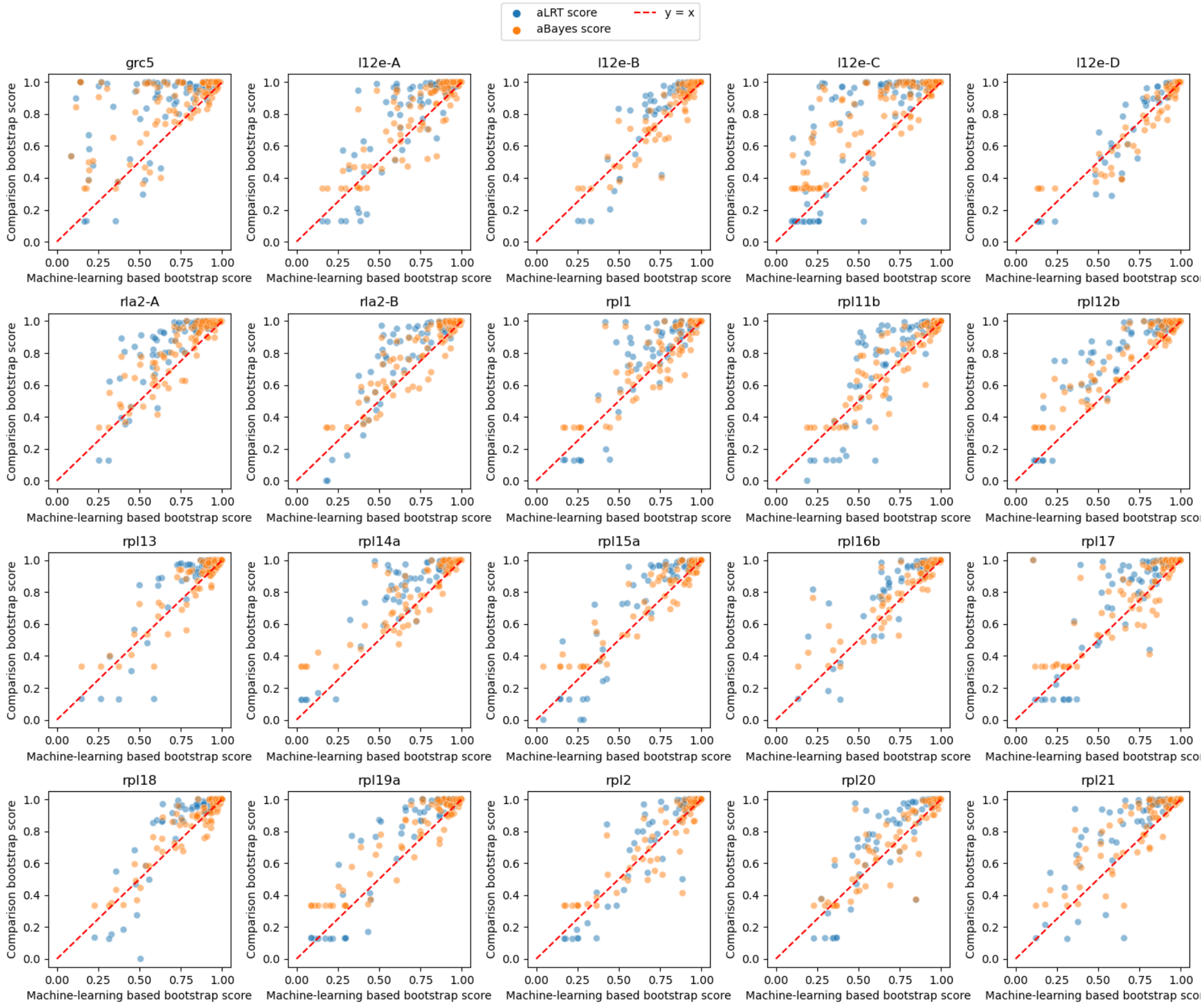

Comparison of the machine-learning bootstrap prediction with aBayes score and aLRT score implemented in IQTREE. Comparison is performed on 20 Metazoa empirical protein MSAs. The red dashed line represents the diagonal x=y line.

D.

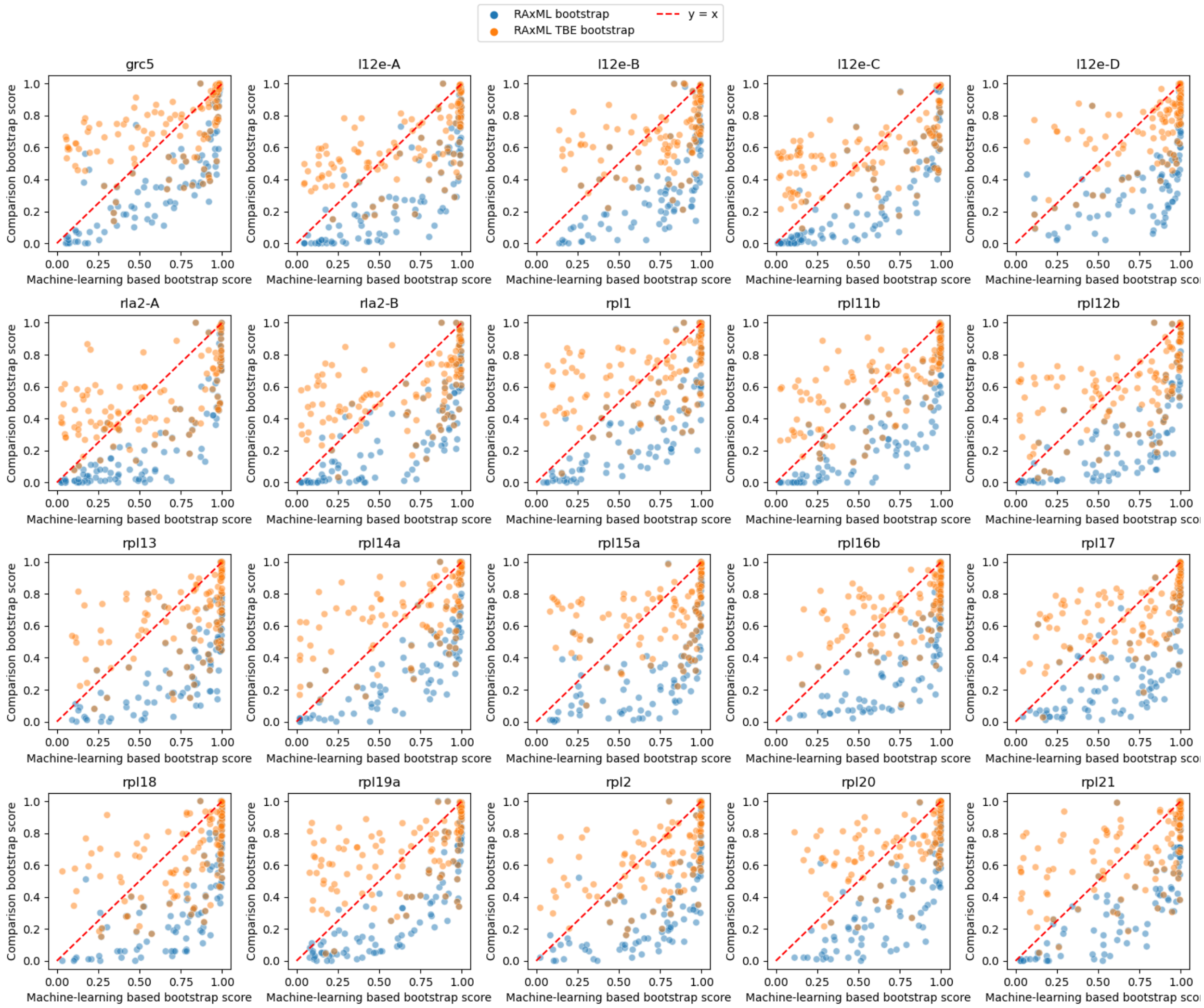

Comparison of the machine-learning bootstrap prediction with the standard bootstrap score and TBE bootstrap score implemented in RAxML-NG. Comparison is performed on 20 Metazoa empirical protein MSAs. The red dashed line represents the diagonal x=y line.
